# Long-term follow-up of herpetic non-necrotizing retinopathy with occlusive retinal vasculitis and neovascularization

**DOI:** 10.1186/s12348-015-0038-z

**Published:** 2015-02-28

**Authors:** Kim Albert, Maureen Masset, Sabine Bonnet, François Willermain, Laure Caspers

**Affiliations:** Department of Ophthalmology, Centre Hospitalier Universitaire Saint-Pierre, Rue Haute 322, 1000 Brussels, Belgium; Centre Hospitalier Universitaire Brugmann, Place Arthur Van Gehuchten 4, 1020 Brussels, Belgium; Department of Ophthalmology, Centre Hospitalier Regional de la Citadelle, Boulevard du 12ème de Ligne 1, 4000 Liège, Belgium

**Keywords:** Uveitis, Herpes, Virus, Simplex, Zoster, Retinal necrosis, Polymerase chain reaction, Non-necrotizing, Vasculitis

## Abstract

**Background:**

Herpetic necrotizing retinitis is a well-recognized entity. A few cases of herpetic non-necrotizing retinitis were previously reported.

**Findings:**

We retrospectively report two cases of herpetic non-necrotizing retinopathy with a long follow-up. A 19-year-old woman presented with a bilateral diffuse occlusive retinal vasculitis, peripheral neovascularization, and no signs of retinal necrosis. Long-lasting immunosuppressive treatment failed to control the vasculitis until herpes simplex virus type 1 (HSV1) was demonstrated by polymerase chain reaction (PCR) in the aqueous. Acyclovir was then added and immunosuppressive tapered and eventually stopped resulting in a resolution of vasculitis. Only two relapses occurred during the next 6 years and responded rapidly to oral acyclovir.

An 11-year-old boy presented with unilateral scar of stromal keratitis, severe vitritis, and optic disc neovascularization, followed 6 weeks later by peripheral occlusive retinal vasculitis. Therapeutic and diagnostic vitrectomy was performed, and PCR was found to be positive for varicella zoster virus (VZV) in a vitreous specimen. The inflammation responded to oral acyclovir therapy. Recurrence of anterior uveitis with iris depigmentation occurred 4 months after treatment was arrested. After 4 years, he presented again with a recurrence of anterior inflammation and cystoid macular edema (CME). No sign of inflammation was seen for the next 10 years.

**Conclusions:**

These rare cases support the possible role of herpes viruses (HSV or VZV) in occlusive vasculitis without retinal necrosis. PCR was useful to raise the diagnosis and to adapt the treatment. A good response was obtained on oral antiviral therapy.

## Findings

### Introduction

Approximately one third of all uveitis cases are associated with infectious agents [1], and another third remains with an unknown etiology [2]. Polymerase chain reaction (PCR) on intraocular fluids is a reliable and highly sensitive test for the diagnosis of infectious uveitis, including cytomegalovirus (CMV), herpes simplex virus (HSV), and varicella zoster virus (VZV) [3,4].

Several types of herpetic posterior uveitis have been described. The acute retinal necrosis (ARN) syndrome is characterized by peripheral retinal necrosis, retinal arteritis, and a prominent inflammatory reaction in the vitreous and anterior chamber of apparently immunocompetent patients [5]. Progressive outer retinal necrosis syndrome is also a herpetic retinal necrosis with minimal vitreous inflammation occurring in severely immunocompromised patients [6]. A few cases of herpes virus-related non-necrotizing posterior uveitis have been described. Bodaghi et al. described five patients with posterior uveitis unresponsive to steroids and a positive PCR for herpes viruses (HSV or VZV) in the aqueous. The disease did not respond to conventional therapy with systemic corticosteroids and/or immunomodulatory therapy, but favorable response was achieved when therapy was switched to systemic antiviral medication [7].

We report two new cases of herpetic non-necrotizing retinopathy with long follow-up.

### Patients and methods

Patient files were retrieved and retrospectively reviewed. Ophthalmic and systemic data were collected in accordance with the ethic committee of the hospital.

#### Case 1

A 19-year-old female was referred in September 1993 for the management of an intravitreal hemorrhage in the right eye, retinal neovascularization of the left eye, and bilateral retinal vasculitis.

**Figure 1:**
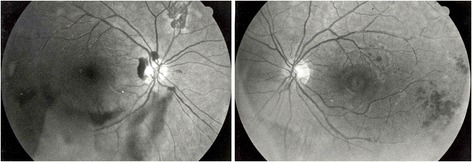
**Patient 1: at presentation.** Intraretinal hemorrhages and periphlebitis in both eyes. The right eye shows a diffuse intravitreal hemorrhage and a chorioretinal scar nasally.

**Figure 2:**
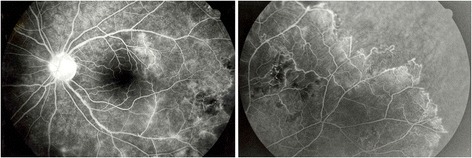
**Patient 1: fluorescein angiography at presentation.** Diffuse vasculitis and peripheral ischemia of the left eye.

Visual acuity (VA) was 20/20 in both eyes. Anterior segment examination was normal. Fundus examination showed severe bilateral occlusive vasculitis (periarteritis and periphlebitis) with intraretinal hemorrhages and peripheral neovascularization. A diffuse vitreal hemorrhage was observed in the right eye while it was more preretinal in the inferior part of left eye. There was no sign of retinal necrosis (Figures 1 and 2).

**Figure 3:**
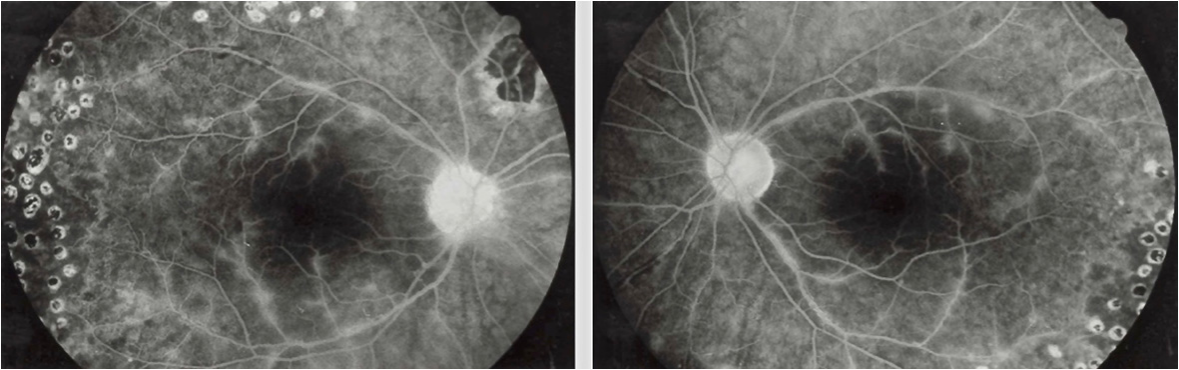
**Patient 1: fluorescein angiography during immunosuppressive treatment.** Retinal photocoagulation was performed in both eyes. Despite immunosuppressive treatment, vasculitis remained active in both eyes.

All known causes of ocular vasculitis as sarcoidosis, presumed tuberculosis-related vasculopathy, systemic lupus erythematosus, antiphospholipid syndrome, Wegener’s granulomatosis, Susac’s syndrome, multiple sclerosis, Behçet’s disease, and syphilis were excluded by an extensive workup. An extensive blood analysis, chest x-ray, tuberculin skin test, bronchoalveolar lavage, and gallium scan were performed, but all results returned negative. A diagnosis of bilateral idiopathic occlusive vasculitis was raised. Oral corticosteroid therapy was administered, and laser photocoagulation of the ischemic zones was performed. Therapeutic response remained poor. Therefore, immunosuppressive agents were added. However, despite a strong immunosuppressive treatment, multiple recurrences of occlusive vasculitis occurred with recurrent hemorrhages in the posterior pole, mild vitritis, and rare episodes of anterior chamber inflammation. New ischemic areas were treated with additional laser photocoagulation. After 3 years follow-up, she developed a cystoid macular edema (CME) in the right eye with VA of 20/25; vision of the left eye remained 20/20. A therapeutic vitrectomy was performed in 1996. Despite this treatment, she continued to develop vasculitis, progressing towards the posterior pole (Figure 3). In 1998, at recurrence of vasculitis associated with anterior cells, an anterior chamber paracentesis was performed, demonstrating HSV 1 by PCR in two aqueous specimens.

**Figure 4:**
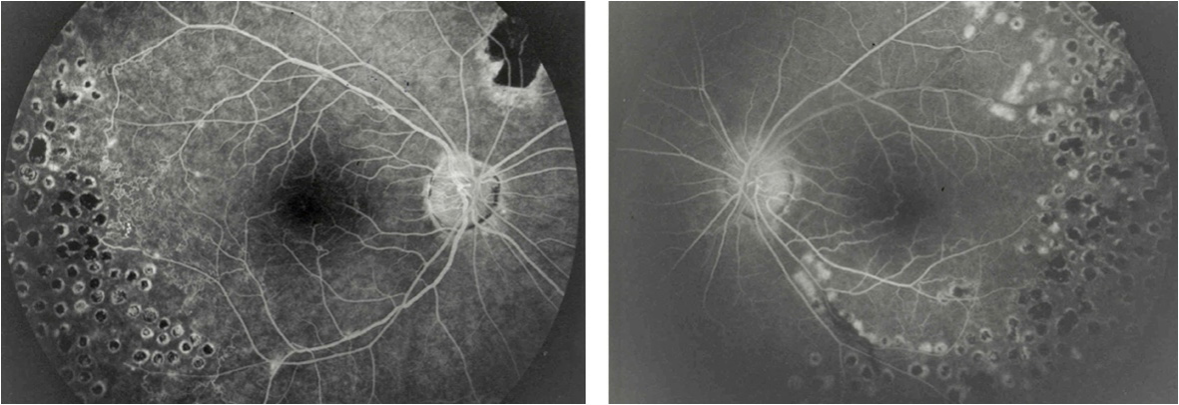
**Patient 1: fluorescein angiography after antiviral treatment.** No signs of vasculitis in both eyes after antiviral treatment.

Acyclovir therapy was initiated at the dosage 5 × 800 mg daily, tapered, and maintained for 7 months, and immunosuppression was tapered and then arrested. A rapid healing of the vasculitis was obtained (Figure 4). Two relapses of retinal vasculitis were reported about 2 and 4 years after the end of oral acyclovir. Both relapses were treated with oral acyclovir alone (800 mg, 5/day) for 3 weeks, with rapid resolution of the vasculitis and no recurrence until the end of the follow-up in November 2004. Final VA was 20/20 in both eyes.

#### Case 2

**Figure 5:**
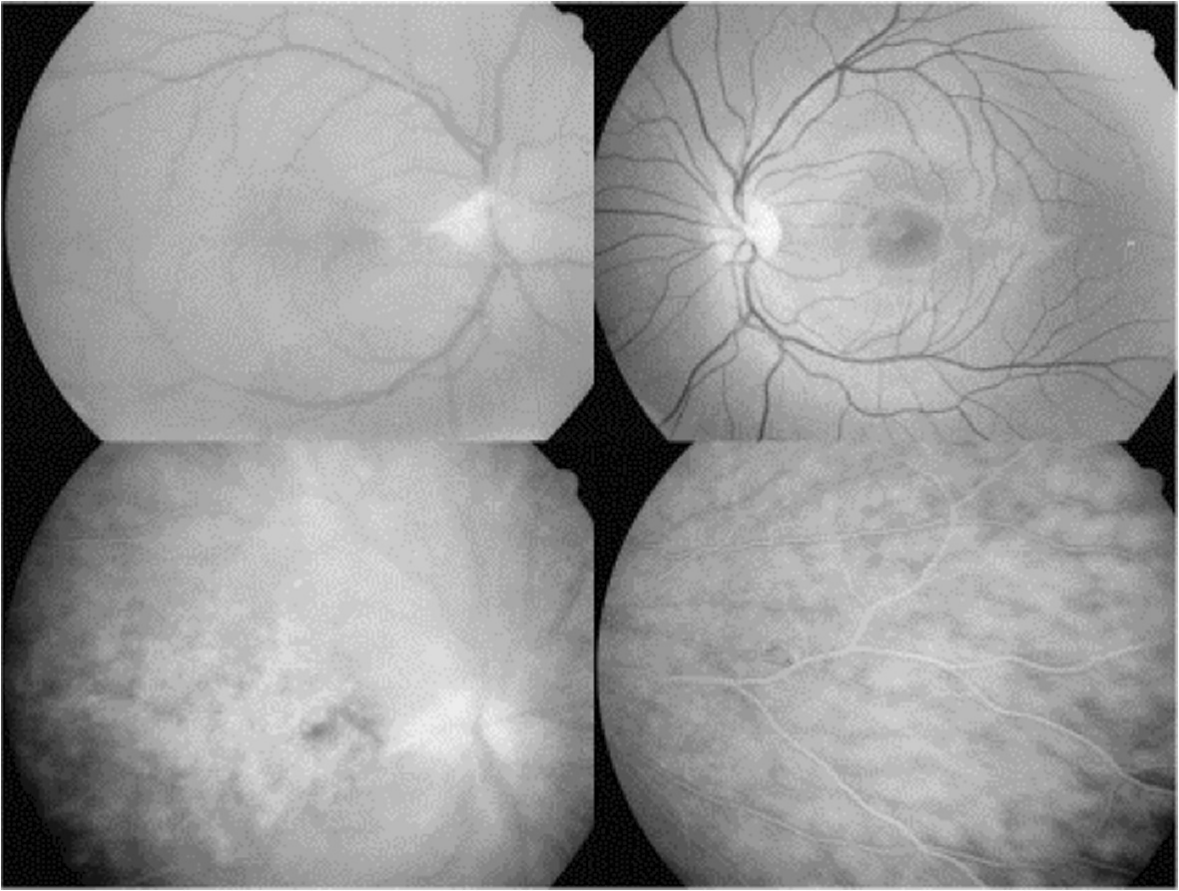
**Patient 2.** Top left: red free photograph of the right eye with residues of the fibrovascular membrane from the optic nerve partially removed during vitrectomy; top right: red free photograph of the unaffected left eye; down: fluorescein angiography of the right eye showing leakage of dye from the optic disc and the posterior and peripheral inflamed retina.

In 1999, an 11-year-old boy presented with a unilateral inferior occlusive retinal vasculitis, optic disc neovascularization, and severe vitritis, limiting the VA of the right eye to 20/50 at presentation. Slight anterior inflammation with scars of endothelial and deep stromal keratitis and inferior pigmented keratic precipitates (KPs) were also observed in the same eye. Six weeks later, he developed inferior retinal neovascularization and VA dropped to 20/200. Because the patient had a tetralogy of Fallot, the surgeon suspected an endogenous bacterial endophthalmitis, and a diagnostic and therapeutic vitrectomy was performed with section of the fibrovascular membranes and photocoagulation of the ischemic non-necrotized peripheral retina. Evaluation of the vitreous specimens revealed the presence of pigmented macrophages without any bacteria. Moreover, PCR performed on the vitreous specimens returned positive for VZV. Oral treatment with acyclovir (5 × 800 mg daily) was administered and VA improved to 20/50. Diffuse leakage of fluorescein was observed from the posterior and peripheral retina as well as the optic disc (Figure 5). Pigmented cells were still present in the anterior vitreous with CME and hypertony during the year following vitrectomy, under topical corticosteroids and oral acyclovir. Oral acyclovir (800 mg, 5/day) was administered for 8 months and then replaced by topical acyclovir and topical steroids for another 8 months. A recurrence of anterior uveitis with posterior iris synechiae and iris depigmentation was observed 4 months later. The patient was lost from follow-up and came back in 2003, after 4 years, with an anterior inflammation with granulomatous inferior KPs of the same eye, slight band keratopathy, depigmentation of the inferior iris, posterior iris synechiae, and posterior subcapsular cataract. The left eye remained normal. The patient was treated with oral acyclovir (800 mg, 5/day) during 3 weeks and long-lasting non-steroidal anti-inflammatory drops. Slight anterior inflammation and CME remained for one more year. No sign of new active inflammation was seen until 2013. The patient had a final VA of 20/200 (right eye) and 20/20 (left eye).

### Discussion

Case reports of non-necrotizing herpetic uveitis have been reported very rarely [7,8]. Bodaghi et al. described five patients with posterior uveitis unresponsive to steroids and whose aqueous aspirates were PCR-positive for HSV or VZV. The clinical presentation consisted of retinal vascular involvement, retinal edema, and papillitis. Anterior inflammation was mild or absent. Posterior segment involvement included features masquerading as Birdshot retinochoroidopathy, Behçet’s disease, or idiopathic retinal vasculitis. All patients had chronic inflammation and no necrotizing forms of herpetic retinopathies that progress as an acute disease (ARN or PORN) [7].

Other cases of atypical variants of ARN have been reported, with a slowly progressive ARN and non-necrotizing forms presenting as vasculitis and/or papillitis or panuveitis [8]. A retinal vasculitis has been described as a complication of primary varicella in an immunocompetent adult [9]. Recently, a case of purely occlusive retinal vasculopathy following varicella zoster infection in an immunocompetent adult, without features of vasculitis or anterior and posterior uveitis, has been published [10]. Chronic herpetic anterior uveitis with focal or multifocal areas of arteriolar sheathing without retinal vascular complications has also been described [11].

We report two new cases of non-necrotizing herpetic retinopathy characterized by occlusive vasculitis with initial neovascularization and a good response to antiviral therapy. The first patient had a history of bilateral recurrent posterior inflammation not responding to steroids and immunosuppressive treatment. This case occurred long before the first case of herpetic non-necrotizing retinopathy was described, which delayed the diagnosis and antiviral treatment. The second patient presented with a unilateral panuveitis. Both patients had early retinal neovascularization, which has not been described previously [7].

A herpetic etiology could be confirmed by PCR analysis of ocular fluids: case 1 had a positive PCR for herpes simplex virus type 1 (HSV1) on the aqueous while case 2 had a positive PCR for VZV on the vitreous. Aqueous analysis was contributory in 86.4% of patients with necrotizing viral retinopathies [4]. Bodaghi et al. found that DNA amplification from aqueous could detect VZV and HSV in non-necrotizing herpetic retinopathies [7].

A remission was obtained in both cases after reaching the proper etiological diagnosis and initiating antiviral treatment. However, several recurrences occurred after arrest of antiviral treatment. The first patient showed relapses of vasculitis after 2 and 4 years, with fast improvement on oral acyclovir. The second patient showed two recurrences of ipsilateral anterior uveitis. Recurrent anterior uveitis following healed ARN has been reported [12], but to our knowledge, this is the first case of anterior uveitis recurrence after non-necrotizing retinopathy described in the literature. We had previously another patient with a recurrence of HSV2-related acute anterior uveitis demonstrated by PCR, several years after a HSV2-related ARN also demonstrated by PCR (L Caspers, unpublished data). Other types of uveitis tend to recur after an ARN episode [12,13]. Even multiple occurrences of ARN separated over long periods can rarely happen in the same eye [14].

Long-standing preventative antiviral therapy could be considered for such patients who present with recurrences of intraocular inflammation.

Our two cases confirm that herpes virus may cause non-necrotizing retinopathy with occlusive vasculitis with early retinal neovascularization. These cases point out the importance of ruling out all infectious, including viral, causes in vasculitis, even in the absence of retinal necrosis, before initiation of immunosuppressive therapy and/or when patients do not respond to immunosuppressive treatment. These two cases are, by our knowledge, the first cases of long-term follow-up of non-necrotizing retinopathy.

### Consent

Written informed consent was obtained from the patients for the publication of this report and any accompanying images.

## Abbreviations

ARN: acute retinal necrosis
CME: cystoid macular edema
CMV: cytomegalovirus
HSV: herpes simplex virus
KPs: keratic precipitates
PCR: polymerase chain reaction
VA: visual acuity
VZV: varicella zoster virus
